# Multimodality treatment in recurrent/metastatic squamous cell carcinoma of head and neck: current therapy, challenges, and future perspectives

**DOI:** 10.3389/fonc.2023.1288695

**Published:** 2024-01-04

**Authors:** Sergio Pannunzio, Armando Di Bello, Denis Occhipinti, Alessandro Scala, Gloria Messina, Giustina Valente, Michela Quirino, Mariantonietta Di Salvatore, Giampaolo Tortora, Alessandra Cassano

**Affiliations:** ^1^ Oncologia Medica, Fondazione Policlinico Universitario Agostino Gemelli Istituto di Ricovero e Cura a Carattere Scientifico (IRCCS), Roma, Italy; ^2^ Comprehensive Cancer Center, Fondazione Policlinico Universitario Agostino Gemelli, IRCCS, Rome, Italy

**Keywords:** head and neck, squamous cell carcinoma, recurrent, metastatic, multimodality treatment, surgery, radiation therapy, chemotherapy

## Abstract

Squamous cell carcinoma of the head and neck is a complex group of diseases that presents a challenge to the clinician. The prognosis in the recurrent/metastatic disease is particularly dismal, with a median survival of approximately 12 months. Recently, the personalized and multimodal approach has increased prognosis by integrating locoregional strategies (salvage surgery and stereotactic radiotherapy) and systemic treatments (chemotherapy, immunotherapy, and target therapy). Malnutrition is a significant clinical problem that interferes with dose intensity, and thus, feeding supplementation is critical not only to increase the quality of life but also to improve overall survival. With this review, we want to emphasize the importance of the multidisciplinary approach, quality of life, and nutritional supportive care and to integrate the latest updates of predictive biomarkers for immunotherapy and future therapeutic strategies.

## 1 Introduction

Worldwide, head and neck squamous cell carcinoma (HNSCC) accounts for 900,000 cases and 400,000 deaths annually and is the sixth most common cancer worldwide (1). The incidence varies across the different areas of the globe and has a high prevalence in Eastern Asia. Approximately 75%–85% of HNSCC is due to tobacco use and alcohol consumption, although human papillomavirus (HPV) infection as a cause of oropharyngeal cancer (OPC) is increasing (2). In the United States, approximately 71% of OPC cases are attributed to HPV (3).

Patients with HPV+ oropharyngeal cancers have a better prognosis than patients diagnosed with HPV-negative disease. The genomic features of HNSCC are very complex and include some driver mutations that might be suitable for targeted therapy, among them HRAS and PI3KCA. As we will discuss in the following paragraphs, many are under investigation (4).

Approximately 10% of patients have distant metastases at diagnosis, while 20%–30% will develop them during the course of the disease. At the same time, patients with locally advanced disease at diagnosis (approximately 2/3 of patients) will develop locoregional recurrence at 2 years in 50% of cases, and 20%–30% of them will also develop distant metastases (2).

In general, the prognosis of these recurrent/metastatic (R/M) patients is poor, with a median overall survival between 6 and 15 months (2).

During the last 30 years, the best therapy for metastatic HNSCC was based on platinum-based poly-chemotherapy with a median overall survival (OS) of 7 months, until 2008 when the EXTREME trial demonstrated a benefit in OS with the addition of cetuximab with platinum salts and 5-fluorouracil (5-FU). Recently, the results of CheckMate 141 and the subsequent KEYNOTE-048 established the role of immunotherapy in the treatment of these patients.

With this review, we want to analyze the current clinician’s weapons against HNSCC in the recurrent/metastatic setting, focusing particularly on immunotherapy and future perspectives.

## 2 Systemic management

The choice of treatment should be based on the evaluation of clinical and molecular parameters: the first includes patients naive to systemic treatments, patients previously treated with adjuvant therapies, the burden of disease (locoregional *vs.* metastatic), local disease recurrence, symptomatic disease, risk of acute complications, Performance Status (PS), platinum-resistant *vs.* platinum-sensitive disease, weight loss, active smoking habit, and significant comorbidities. The second includes HPV-related oropharyngeal disease and PD-L1 expression (5). Moreover, patients with these diseases should be referred to high-volume centers where cases should be discussed in multidisciplinary teams (6).

### 2.1 Naive patients to systemic treatments

According to the cancer-immunity cycle proposed by Chen, Coukos, and Mellman, anticancer activity is modulated by the immune cells, at first with cancer immune recognition, then with an adaptive immune response, and finally with cancer cell elimination. Every step of this process represents a potential target for treatment and strategies to reduce the immune escape phenomenon. Nowadays, multiple predictors and prognostic factors are identified, but only PD-L1 is predictive of the response of immunotherapy (7).

Following this evidence, the standard scenario of medical treatment of metastatic/recurrent naive patients has been enriched by the results of the KEYNOTE-048 phase III trial. In this study, patients were randomized in one of the three following arms: pembrolizumab alone *vs.* pembrolizumab + platinum + 5-FU *vs.* cetuximab + platinum + 5-FU (EXTREME regimen). Patients were stratified according to PD-L1 expression, P16 status, and performance status of Eastern Cooperative Oncology Group (PS ECOG) 0-1. The primary endpoints were OS and progression-free survival (PFS) with the intention to treat (ITT) population (8).

The results of the final analysis suggested that the use of pembrolizumab in PD-L1-positive R/M HNSCC, either as monotherapy or in combination with chemotherapy, was preferred to treatment with EXTREME schedule, considered the standard of care from 2008 to 2019. In particular, the pembrolizumab plus chemotherapy regimen significantly increased OS compared with the EXTREME schedule (13.0 months *vs.* 10.7 months, HR = 0.71; 95% CI, 0.59 to 0.85; p = 0.00008) in the overall population. Objective response rate (ORR), PFS, and incidence of adverse events were similar in the two arms (ORR 36.3% and 36.3%, PFS 4.9 and 5.3 months, grade 3 adverse events (AEs) 71.7% versus 69.3%).

Consistent with expectation, the OS in the population treated with pembrolizumab as monotherapy *vs.* EXTREME regimen was superior in neoplasms with high PD-L1 expression: patients with combined positive score (CPS) ≥ 20 had a median OS of 14.9 months *vs.* 10.8 months (HR = 0.61; CI, 0.46 to 0.81), while patients with CPS ≥ 1 had a median OS of 12.3 months *vs.* 10.4 months (HR = 0.71; CI, 0.61 to 0.89). Pembrolizumab as monotherapy in the overall population did not show an advantage in survival but was not inferior: 11.5 months *vs.* 10.7 months (HR = 0.81; CI, 0.68 to 0.97). Pembrolizumab alone did not improve PFS or ORR compared with cetuximab–chemotherapy (ORR was 23.3% versus 36.1% and 19.1% versus 34.9% in the CPS ≥ 20 and CPS ≥ 1 groups, respectively). The duration of response (DOR), investigated as an exploratory endpoint, in the pembrolizumab alone group with CPS ≥ 1 was approximately 2 years (9).

The 5-year OS rate for pembrolizumab *vs.* EXTREME was 19.9% *vs.* 7.4% in CPS ≥ 20, 15.4% *vs.* 5.5% in CPS ≥ 1, and 14.4% *vs.* 6.5% in the total population. The 5-year OS rate for pembrolizumab + chemotherapy *vs.* EXTREME was 23.9% *vs.* 6.4% in CPS ≥ 20, 18.2% *vs.* 4.3% in CPS ≥ 1, and 16.0% *vs.* 5.2% in the total population (8).

In *post-hoc* subgroup analysis in the PD-L1 CPS < 1 for pembrolizumab alone versus cetuximab–chemotherapy, the median overall survival was 7.9 versus 11.3 months (HR = 1.51), while for pembrolizumab–chemotherapy versus cetuximab–chemotherapy, the median overall survival was 11.3 versus 10.7 months (HR = 1.21). Although not prespecified in the design of the study, the PD-L1 CPS 1-19 subgroup obtained a median OS of 10.8 for pembrolizumab monotherapy versus 10.1 months of the cetuximab–chemotherapy subgroup (HR = 0.86). In the pembrolizumab–chemotherapy arm, the median OS was 12.7, and in the cetuximab–chemotherapy arm, it was 9.9 months (HR = 0.71) (10).

Following these results, pembrolizumab monotherapy can be considered starting from high PD-L1 expressions with CPS ≥ 1 but should be preferred in patients with CPS ≥ 20 and in cases where the disease is not progressing quickly. In contrast, the combination (pembrolizumab plus chemotherapy) could be the best option in patients symptomatic or with rapidly progressing disease, when rapid tumor shrinkage is required, regardless of PD-L1 expression.

To date, pembrolizumab is approved by the Food and Drug Administration (FDA) in combination with chemotherapy, independently of PD-L1 expression, and as monotherapy for patients with PD-L1-expressing tumors (CPS ≥ 1); on the contrary, the European Medicines Agency (EMA) has approved pembrolizumab with or without chemotherapy in patients with CPS ≥ 1, thus designating patients with CPS < 1 for chemotherapy-only regimens.

In consideration of the potential activity of immunotherapy in patients with metastatic/recurrent disease, the efficacy of the ipilimumab–nivolumab combination was investigated in CheckMate 651; in this phase III study, nivolumab plus ipilimumab did not result in a statistically significant improvement in OS versus EXTREME in platinum-eligible R/M HNSCC. The primary endpoints were OS in the all randomly assigned and PD-L1 CPS ≥ 20 populations. The median OS was 13.9 months with nivolumab plus ipilimumab versus 13.5 months with EXTREME in the all randomly assigned population (HR = 0.95; CI, 0.80 to 1.13; p = 0.4951); it was 17.6 months versus 14.6 months in the CPS ≥ 20 population (HR = 0.78; CI, 0.59 to 1.03; p = 0.0469) and did not reach statistical significance in either two primary endpoints. Safety with nivolumab plus ipilimumab was favorably compared with EXTREME: grade 3/4 treatment-related adverse events occurred in 28.2% versus 70.7%, respectively (11).

Although the study did not reach the endpoints, it is notable that the population with CPS ≥ 20 obtained a median OS that was close to statistical significance (HR = 0.78, p = 0.0469) and could be considered clinically meaningful; the objective response rate was 34%, nearly overlapping the control arm (36%), and the median duration of response of 32.6 months (*vs.* 7.0) is the longest recorded in this stage disease. In addition, in the CPS ≥ 20 population, the median time to symptom deterioration was 16.7 *vs.* 7.6 months (11). Finally, we should mention that the median OS in the EXTREME arm in the intention-to-treat population was higher (13.5 months) than the historically reported time of 10.1 months.

The phase II trial CheckMate 714 is underway, which randomized patients to receive nivolumab alone or in combination with ipilimumab in recurrent or metastatic HNSCC (NCT02823574).

In patients with contraindications to immunotherapy or with CPS < 1, the EMA-approved standard first-line treatment remains the EXTREME schedule with cisplatin–5-fluorouracil–cetuximab. In the randomized phase III EXTREME trial, the experimental arm significantly prolonged survival (median 10.1 versus 7.4 months, HR for death = 0.80; 95% CI, 0.64 to 0.9), PFS (median 5.6 versus 3.3 months), and ORR (36% versus 20%) compared with the chemotherapy-only arm (platinum plus fluorouracil) (12).

The use of a taxane as an alternative to 5-fluorouracil may be considered in patients who are not candidates for fluoropyrimidine. Evidence in favor of this combination comes from the phase II non-inferiority B-490 trial that randomized 148 patients to receive cetuximab plus cisplatin with or without paclitaxel (13) and the GORTEC phase II study that randomized 539 patients to receive the (cis)EXTREME scheme for 6 cycles *vs.* the TPEx (platinum–docetaxel–cetuximab) scheme for 4 cycles (14). The study results should be considered negative, as they did not meet the primary endpoint of superiority in OS of the experimental arm (14.5 months *vs.* 13.4 months, HR = 0.89; 95% CI, 0.74 to 1.08; p = 0.23) and did not show statistically significant differences in PFS and ORR. A point in favor of the experimental arm was the better toxicity profile, probably due to the lower number of cycles, lower dose of cisplatin (100 mg/mq *vs.* 75 mg/mq), and systematic granulocyte colony-stimulating factor (G-CSF) primary prophylaxis. Due to these results, the TPEx schedule could be considered in patients who are not candidates for 5-fluorouracil treatment.

The KEYNOTE-B10 is an ongoing single-arm phase IV trial that enrolled 92 patients, previously untreated, to receive pembrolizumab–carboplatin–paclitaxel, regardless of PD-L1. Although data are still immature, and longer follow-up is needed. The ORR was 43% (95% CI, 32 to 54), and the median OS showed a positive trend with 12.1 months (NCT04489888).

The combinations of platinum and taxanes were demonstrated to be active either in phase II or in phase III studies, but they were not superior to the platinum–fluorouracil combinations, with overlapping response rates and survival (15) (16).

### 2.2 Non-platinum-based regimens

Other combinations may be useful in patients who are not candidates for platinum-based chemotherapy.

The SWOG trial was a single-arm phase II study that evaluated 57 patients with metastatic or recurrent head and neck cancer, with the combination of gemcitabine (3,000 mg/mq) plus paclitaxel (150 mg/mq) administered biweekly, and was associated with a 28% ORR (17).

Median PFS and OS were 4 and 8 months, respectively. However, there are no data about the superiority of this combination in comparison to single-agent taxane therapy. In an open-label phase II trial, the combination of weekly paclitaxel and cetuximab showed 54% ORR, with median PFS and OS of 4 and 8 months, respectively (18).

As we discussed in the Quality of Life section, many patients with HNSCC are frail, and many of them are ineligible for cisplatin for several reasons: renal failure, cardiologic comorbidities, age > 70 years, and PS ECOG > 2. In this category of patients, there is no strong evidence for an alternative regimen to cisplatin. A retrospective study demonstrated the efficacy and safety of weekly carboplatin AUC 2 in combination with weekly paclitaxel in patients ineligible for cisplatin (19).

These results led to investigating the combination of durvalumab with weekly paclitaxel and carboplatin AUC 2 in frail patients ineligible for cisplatin in a single-arm phase II study (FRAIL-IMMUNE). This study met its primary endpoint by achieving a median OS of 18 months; 20.4% of patients experienced a grade G3 adverse event, which has a better toxicity profile than KEYNOTE-048 (in the pembrolizumab–chemotherapy arm, grade 3–4 adverse events were 47%). These results need to be confirmed in a comparative phase III trial (20).

### 2.3 Platinum refractory

Platinum refractory refers to all patients who relapse in less than 6 months after the end of platinum treatment. In these patients, the prognosis is poor.

Both nivolumab and pembrolizumab are recommended by the National Comprehensive Cancer Network (NCCN) guidelines, based on the results of two phase III trials: CheckMate 141 and KEYNOTE-040. Both studies enrolled patients regardless of PD-L1 expression, showing, however, a better effect of both agents in the PD-L1-positive population (21).

The CheckMate 141 trial demonstrated the superiority of nivolumab in comparison with standard single-agent treatments (docetaxel, methotrexate, or cetuximab) in terms of OS, which was the primary endpoint: 7.5 months *vs.* 5.1 (HR = 0.70; 97.73% CI, 0.51 to 0.96; p = 0.01). The treatment-related events of grade 3 or 4 occurred in 13.1% of the patients in the nivolumab group versus 35.1% of those in the standard treatment (22).

In the KEYNOTE-040 phase III study, which compared pembrolizumab *vs.* standard of care, the median OS was higher but not statistically significant (8.4 versus 6.9 months; HR = 0.80, 0·65–0·98; nominal p = 0.0161). In the subgroup analysis of patients with PD-L1 expression of more than 50% (tumor proportion score (TPS)), the median OS was 11.6 versus 7.9 months (HR = 0.54) (23). Pembrolizumab was approved by the EMA only for patients with PD-L1 ≥ 50%.

Several trials attempted to evaluate the efficacy of dual checkpoint-inhibitor (IO-IO) combination therapy. The results seem to suggest that the combination is not characterized by a synergistic activity. In 2019, the randomized phase II study CONDOR enrolled 267 patients with progression during or after first-line treatment with platinum-based for R/M disease and with absent or low PD-L1 expression (<25% TC). Patients were randomized in a 2:1:1 ratio to receive combination therapy with durvalumab/tremelimumab (IgG2 antibody to CTLA-4) versus durvalumab monotherapy versus tremelimumab monotherapy. This study did not prove the hypothesis that tremelimumab combined with durvalumab could exert a synergistic therapeutic effect, in terms of RR, in this population with low or no expression of PD-L1 (24).

The phase III EAGLE trial enrolled patients with relapsed/metastatic disease progressing during or after first-line platinum-based treatment; they were randomized to receive 1:1:1 durvalumab, durvalumab plus tremelimumab, or standard therapy (SoC) (cetuximab, taxanes, methotrexate, or a fluoropyrimidine). No benefit in terms of overall survival was observed either in the durvalumab arm versus SoC (HR = 0.88; 95% CI, 0.72 to 1.08; p = 0.20) or in the durvalumab versus tremelimumab arm versus SoC (HR = 1.04; 95% CI, 0.85 to 1.26; p = 0.76); OS at 12 months was 37% for durvalumab, 30.4% for combination arm, and 30.5% for SoC (25).

With these results, current international guidelines do not recommend IO-IO combination therapy.

**Figure 1:**
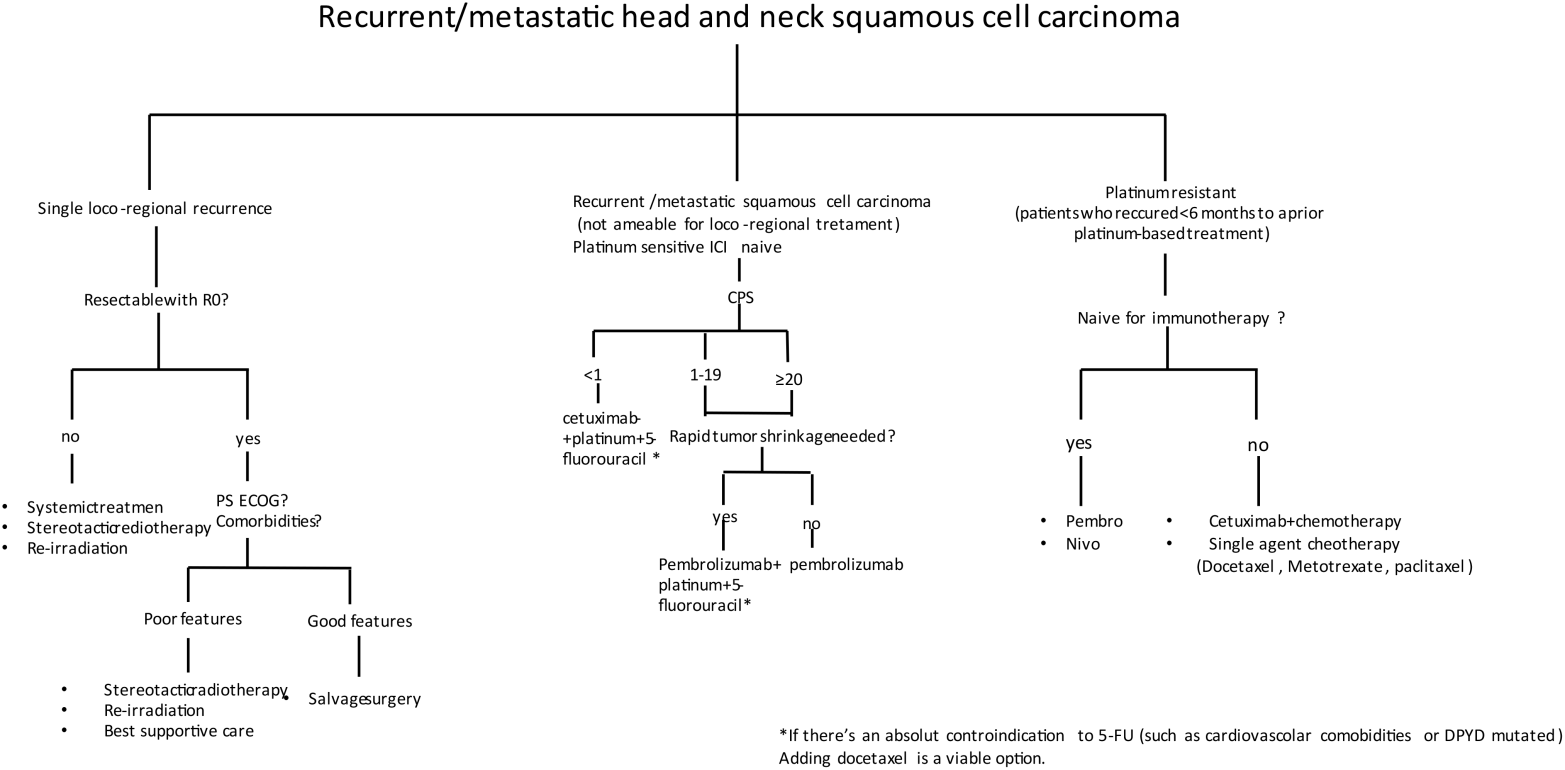
Decision-making algorithm in the recurrent/metastatic head and neck squamous cell carcinoma (R/M HNSCC).

In patients who received immunotherapy in the first line, no standard of care exists; single-agent chemotherapy could be proposed, such as docetaxel, methotrexate, paclitaxel, or capecitabine. Until now, there are no data about the best option after immunotherapy from randomized trials, while there are few published retrospective data regarding combinations of chemotherapy after upfront immune checkpoint inhibitor (ICI) demonstrating intriguing response rates both with platinum- and 5-FU-based doublet (26) or cetuximab-based (27) therapies (**Figure 1**). Several prospective studies beyond the progression of ICI are underway (**Table 1**).

**Table 1:** Ongoing selected studies beyond progression on ICI.

Clinical trial.gov NCT identifier	Study type	Regimen	Study population
NCT05721443	Open-label, single-arm, phase II study	Cetuximab plus dalpiciclib (CDK4/6 inhibitor)	HPV-negative, PD-1-resistant R/M HNSCC
NCT05063552	Phase II/III study	Standard therapy (CT + cetuximab) *vs.* CT + atezolizumab *vs.* atezolizumab + bevacizumab	R/M HNSCC progressed on 1st-line pembrolizumab
NCT05054439	Multicenter, open-label, phase II study	SI-B001 (anti-EGFR/HER3 Ab) plus paclitaxel	R/M HNSCC progressed on prior 1st or 2nd line with anti-PD-1 + platinum-based CT (non-nasopharyngeal carcinoma)
NCT05283226	Multicenter, open-label, single-arm, phase II study	Oral NRC-2694-A (anti-EGFR small-TKI) plus paclitaxel	PD-1-resistant R/M HNSCC
NCT05751512	Multicenter, open-label, phase III	MRG003 (anti-EGFR ADC) *vs.* cetuximab/methotrexate	R/M HNSCC progressed on prior 1st or 2nd line with anti-PD-1 + platinum-based CT

HNSCC, head and neck squamous cell carcinoma; CT, chemotherapy; ICI, immune checkpoint inhibitor; Ab, antibody; ADC, antibody–drug conjugate; TKI, tyrosine kinase inhibitor.

## 3 Salvage surgery

Locoregional recurrence, with no other evidence of metastasis, can be treated in a curative intent with salvage surgery. Time to first recurrence was the single most important factor affecting survival.

A recent meta-analysis conducted by Bulbul et al. analyzed 15 studies (a large part retrospective studies) comparing salvage surgery versus non-surgical treatments in patients with locoregional recurrence of HNSCC including tumors of the oral cavity, pharynx, and larynx. This meta-analysis demonstrated a consistent 5-year OS advantage of surgery compared to non-surgical treatments, with an HR of 0.25 (28). In a previous meta-analysis of 32 studies, with a total of 1,080 patients, Goodwin et al. showed a 5-year OS benefit of 39% (29).

The site of the primary tumor and its radical resection are important prognostic factors. The reason can be attributed to the relationship of anatomical structures that are critical to ensure the operability of the tumor.

Recurrences of hypopharynx tumors are characterized by poor prognosis in relation to the anatomical structures involved in the field of the primary tumor; on the contrary, recurrences of laryngeal tumors are associated with a better prognosis, with 70% of OS at 5 years after salvage resection that may include radical laryngectomy or conservative surgical treatment (30).

The greatest challenge of the multidisciplinary team concerns the correct selection of patients suitable for salvage surgical resection. In Lupato’s meta-analysis, 25 studies were included, with a total of 1,280 patients undergoing salvage surgery. The pre-surgical prognostic factors associated with a statistically significant worsening were disease-free interval <12 months (HR = 1.91), age > 60 years (HR = 1.82), and stage III–IV at diagnosis (HR = 1.5). Positive surgical margins (HR = 2.34), extra-capsular lymph node extension (HR = 4.31), and complications after surgery (HR = 1.91) were correlated with a post-surgical worse prognosis (31). Post-surgical complications are a huge problem in these patients: in a systematic review of 3,293 patients who underwent laryngectomy after the failure of radio-chemotherapy complication rates were 67.5%, including the most common fistulas with an incidence of 28.9% (32).

In a patient who has a single metastasis or with a single locoregional recurrence, is it better to have salvage surgery or “curative” radiation therapy? There are no randomized clinical trials, and the only available evidence is from retrospective studies; the data seem to show that salvage surgery prolongs locoregional failure (LRF) and OS (33). The goal to be achieved in salvage surgery is to obtain R0. Patients with gross residual disease after surgery had LRF at 2 years similar to that of permanently treated patients (47.4% *vs.* 46.3) (34).

In patients who cannot be treated with salvage surgery, radiation therapy for “curative” purposes remains an option in selected cases with good PS and a recurrence-free interval (35). Adjuvant radiotherapy is an option after salvage surgical treatment, especially in high-risk patients (36). A phase III study attempted to answer the question of whether chemotherapy (hydroxyurea and 5-FU) should be added with radiotherapy. The study showed an increase in disease-free survival (DFS) but not in OS, at the cost of a consistent increase in toxicity. Therefore, to date, there is no indication for the addition of chemotherapy to radiotherapy in these patients (37).

Recently, immunotherapy has emerged as a potential treatment for this disease scenario. The ADJORL study evaluated the use of nivolumab immunotherapy after salvage surgery treatment. It is a non-randomized phase II study that enrolled 57 patients who relapsed after previous radiotherapy treatment and were subsequently treated with curative intent with salvage surgery. After a 2-year follow-up, DFS was 46.6%, and OS was 67.3% (38).

In conclusion, in a patient with locoregional recurrence without further metastasis or with a single metastatic site, with good PS ECOG, when R0 is technically feasible, salvage surgery should be taken as the first treatment option. In case the patient cannot receive surgical treatment due to poor general condition, or comorbidities, or when R0 surgery is not possible, reirradiation is a viable option.

## 4 Radiotherapy in recurrent/metastatic head and neck cancer

Technological and clinical advances achieved in the field of radiation therapy (RT) have improved the balance between tumor control and its effects on normal tissue (39).

Curative-intent radiation therapy is delivered with doses from 6,000 to 7,000 cGy divided into 180- to 200-cGy fractions and is frequently combined with chemotherapy. The most frequent toxicities with these regimens are mucositis, dysphagia, xerostomia, dysgeusia, and radiation dermatitis. In contrast, palliative regimens try to lower the radiation dose to below the threshold for severe side effects in order to maximize the balance between risk and benefits (40).

### 4.1 Palliative radiation regimens

To date, there are no standard recommendations from guidelines on which regimen to adopt, and the choice is often at the discretion of the radiotherapist. One possible treatment regimen is the “QUAD shot”, which consists of the administration of 4 Gy over 2 days in two fractions per day. Patients could receive up to 2 additional cycles if they have not demonstrated tumor progression at the time of follow-up. In a phase II study, an ORR of 53% was observed, and 44% of patients had an improvement in quality of life (41). In a retrospective study by Nguyen, a palliative regimen consisting of three fractions of 8 Gy each, given on day 0, day 7, and day 21 for a total of 24 Gy, showed a 40% complete response for symptoms and 50% ORR (42). The AIIMS trial evaluated the use of the short-course regimen of 20 Gy in five fractions, one per week; this schedule relieves difficult physical symptoms for a period of approximately 7 months. Of 505 patients, 37% achieved a partial response (43). “QUAD shot” regimen, 24 Gy in three fractions, or 20 Gy in five fractions allows symptom palliation in patients with symptomatic disease and poor prognosis (less than 4 months), with a reduction of treatment toxicity rate. Patients with an intermediate prognosis (less than a year) who do not have other treatment options may benefit from a conventional palliative regimen (40).

### 4.2 Oligometastatic disease

Selected patients with oligometastatic and oligo-recurrent head and neck cancer may benefit from a therapeutic approach.

In patients with up to five metastatic sites from any primary tumor site, the phase II SABR-COMET trial exhibited improvements in OS (50 *vs.* 28 months, p = 0.006; HR = 0.47) when metastatic sites were treated with stereotactic body radiotherapy (SBRT) (44). Other evidence in selected patients with oligometastatic HNSCC who underwent surgery or SBRT to metastases reported 5-year survival rates of 20%. Given this evidence, in patients with oligometastatic disease and good performance status, a course of 70 Gy in 35 fractions should be considered (45). The OMIT study is a randomized phase II trial evaluating radiotherapy alone versus radiotherapy + chemotherapy in oligometastatic patients. Fifty-nine patients with oligometastatic disease, defined as one to three metastases, were enrolled, and the 1-year OS was almost overlapping (63.4% with SABR-alone *vs.* 61.7% with chemo-SABR); the 1-year PFS rate was decreased. One of the most important data in the study was toxicity, with a clear advantage rate of all grade toxicities in patients receiving SARB-alone (29.4%) versus (94.3%) with chemo-SABR, without quality of life (QoL) deterioration (46).

A single-institution retrospective study reviewed the outcomes of 1,000 consecutive stage III to IVB HNSCC previously treated with radical intent who developed oligometastases. Patients with single metastasis experienced significantly improved OS (25.7 months) *vs.* those with two to four (11.3 months) or five or more metastases (7.5 months) (p = 0.002). Most of these patients underwent local therapy of metastases with either surgery or radiotherapy with definitive intent. In multivariate analysis, the parameters related to survival after distant metastasis treatment included the time to develop metastases, Karnofsky performance status greater than 70, non-oral cavity primary tumor, and a single metastatic lesion (47).

### 4.3 Reirradiation

There are a few data regarding palliative-intent reirradiation; the RTOG 9610 (48) and RTOG 9911 (49) trials assessed curative-intent salvage reirradiation after radio-chemotherapy. The role of reirradiation in the current era of intensity-modulated radiation therapy (IMRT) is not exactly defined. The selection of patients to undergo reirradiation is challenging and needs to be led by a multidisciplinary team. Patients with more than 2 years since their first course of radiation (34) and ECOG performance score of 0 (50) had better outcomes in this sample.

Proton therapy is increasingly used as an accepted form of reirradiation to reduce the complications associated with a second course of radiation. In a single-institution retrospective cohort, Lee et al. found that proton therapy reirradiation (PT-ReRT) may be associated with good survival in patients with recurrent HNSCC, with an aggressive regimen associated with better outcomes. However, surviving patients remain at risk of early and late complications (51). Proton beam treatment (PBT) is supported by data that primarily come from non-randomized institutional reports and a small number of systematic studies, which have demonstrated that PBT is safe in a controlled setting. However, without high-quality prospective comparative data, it is premature to conclude that proton therapy has been established as superior to other modern radiation techniques such as IMRT (52). Prospective comparative clinical trials are ongoing (NCT03164460).

### 4.4 Immunotherapy and radiotherapy

Several preclinical and clinical studies have elucidated possible mechanisms by which radiotherapy enhances the effect of ICI. Nonetheless, RT works as an *in situ* vaccination promoting tumor antigen cross-presentation and inducing the production of chemokines and cytokines to enhance the local and abscopal antitumor immune response (53).

RT immunosuppressive effects result in the inactivation of approximately 90% of lymphocytes exposed to 3 Gy *in vitro* colony (54). Preoperative RT in oral squamous cell carcinoma has been shown to significantly induce the proliferative activity of CD8+ tumor-infiltrating lymphocytes (TILs), and TILs’ relative radioresistance has been attributed to transforming growth factor (TGF), which is already induced by low-dose RT (55). Nevertheless, RT can increase the concentration of immunosuppressive cells in the HNSCC tumor microenvironment (TME), and the magnitude of this effect seems to depend on RT details: hypofractionated RT increases T-cell tumor infiltration, downregulates intratumoral immunosuppressive vascular endothelial growth factor (VEGF), and leads to a lower increase in myeloid-derived suppressor cells (MDSCs) as compared to conventionally fractionated RT (56). RT can also influence TME increasing cancer stem cells (CSCs) much more in HPV− HNSCC than in HPV+ HNSCC (57).

At the same time, RT causes a dose-dependent increase in major histocompatibility complex (MHC) I expression *in vitro* as well as *in vivo* (58). Furthermore, RT enhances the diversity of PD-1+CD8+ T cells, which are positive predictors of response to anti-PD-1 therapy (59). RT produces free cytosolic DNA, especially in cells with loss of p53 function, which is lost in a majority of HPV− HNSCC (60, 61).

The phase II trial by McBride et al. randomized 62 patients with metastatic HNSCC to nivolumab *vs.* nivolumab + SBRT (3 × 9 Gy). Patients had at least two metastatic lesions: one that could be safely irradiated and one measurable by Response Evaluation Criteria in Solid Tumors (RECIST). The primary endpoint was ORR in non-irradiated lesions. There were no significant differences between nivolumab alone and combination arm in terms of ORR, median PFS, OS at 1 year, or toxicities. In the 56 patients with positive expressions of PD-L1 (TC ≥ 1%), the ORR was higher (50%) compared to that of PD-L1-negative patients (23.5%). HPV-positive patients had a higher ORR (41.9%) compared to HPV-negative patients (20.7%). Although the test for interaction, when evaluated in a multivariate analysis of ORR that included both treatment groups and viral status, was not significant (p = 0.16), the proportion of responding patients with virus-negative disease was higher with nivolumab plus SBRT than with nivolumab alone. According to these data, tumors that are less inflammatory and virus-negative may benefit more from radiotherapy-increased antigen presentation (62).

One of the possible reasons for the failure of this study could be the correct timing of radiotherapy (before, during, or after immunotherapy treatment)?, which still remains a topic of debate; different studies are evaluating sequential radiation treatment (63). Moreover, not all metastatic sites have the same proportion of immunogenicity. Evidence from non-small cell lung cancer (NSCLC) studies has shown that irradiation on liver metastases has stronger immunogenicity than irradiation on lung metastases (64).

Another possible explanation could be in the type of immunotherapy. Evidence demonstrated that anti-CTLA-4 may facilitate a stronger radiation-mediated vaccination effect and deplete myeloid-derived suppressor cells (65).

In conclusion, radiotherapy in combination with immunotherapy is a great topic of scientific research that poses many unsolved challenges, which may be highlighted by more preclinical studies.

### 4.5 The abscopal effect

The therapeutic effect of RT is mediated not only by direct energy deposition to the exposed target but also by the so-called abscopal effect wherein distal lesions respond to the local treatment (66).

Concurrent RT and anti-CTLA-4 antibody therapy successfully induced the abscopal effect in animal trials (67). RT regimens delivered in higher total doses and hypofractionation show no evidence of the abscopal effect despite benefits in tumor control and symptom relief (68), while fractionated RT (3 × 8 Gy or 5 × 6–10 Gy) in combination with anti-CTLA-4 induces a higher abscopal response (69).

Preclinical studies have shown that partial tumor irradiation is not inferior to full-volume irradiation in the same dose. In the non-irradiated section, an increase in CD8+ T-cell concentration was observed. Hemibody irradiation also elicited an abscopal effect, which was comparable to the one observed after whole tumor irradiation (70). Clinical experience appears to support these findings. Seventy-nine patients with metastatic cancers, of which four had HNSCC, received SBRT in various fractionations for two to four metastases followed by pembrolizumab within 7 days after SBRT; at 6 months, there was no difference in local control between fully and partially irradiated lesions (71). Only partially irradiating peritumoral tissue could provide benefits with concurrent immunotherapy, reducing severe damage.

## 5 Quality of life

Patients with head and neck cancers have usually a poor quality of life, compared with patients affected by other neoplasms (72) (73). This is mainly due to the impaired ability to feed related either to anatomical organs involved by neoplasm or to the toxicity of treatments like surgery and high doses of radiotherapy. These patients, in addition to important anatomical limitations, develop depression and psychosocial impairment that frequently are the basis of their disease (74).

Diagnosis is often performed because of pain; for this reason, pain assessment is a key focus of the patient’s evaluation, and standardized measurements should be used to assess pain intensity (75). The clinician can choose treatment according to the needs and type of pain (neuropathic pain, joint pain, general malaise, post-radiation pain, or post-surgical pain) (76).

There are other issues to watch out for, including painful swallowing and mechanical/functional inability to swallow.

Breakthrough pain in patients with head and neck cancers is characterized by a large number of episodes/day and the predictability, particularly with ingestion of food; thus, it is necessary to set up proper pain therapy based on drugs that meet the needs of patients and allows proper feeding (77), avoiding oral drugs and preferring transdermal drugs and nasal fentanyl preparations (78).

Another key issue is the patient’s ability to feed and breathe independently. Patients with head and neck disease are at major risk of developing severe malnutrition and early cachexia, affecting the ability to carry out treatments with a negative impact on prognosis (79). Careful initial screening of higher-risk patients could enable the scheduling of elective percutaneous endoscopic gastrostomy (PEG) or tracheostomy, preventing the onset of dysfunction and reducing complications of emergency surgeries (80).

The safety profile for pembrolizumab monotherapy in KEYNOTE-048 was better than cetuximab–chemotherapy (grade 3–4, 55% *vs.* 83%) and was comparable in the groups receiving chemo-immunotherapy or EXTREME regimen (grade 3–4, 85% *vs.* 83%).

Patients treated with first-line pembrolizumab, pembrolizumab–chemotherapy, or cetuximab–chemotherapy were evaluated according to the European Organisation for Research and Treatment of Cancer (EORTC) 30 quality-of-life (81), EORTC 35-question quality-of-life head and neck cancer-specific modules (82), and EuroQoL five-dimension three-level instruments (EQ-5D-3L) (83) questionnaires.

Patients still enrolled at week 15 who had received first-line pembrolizumab monotherapy or pembrolizumab–chemotherapy had stable health-related QoL (HRQoL). Pembrolizumab or pembrolizumab–chemotherapy versus cetuximab–chemotherapy led to no clinically meaningful difference in EORTC QLQ-C30 global health status (GHS)/QoL, functioning, and symptom scores (84).

Using the same questionnaires, in the KEYNOTE-040 patient’s cohort, it was shown that in patients treated with pembrolizumab, the median time to deterioration in GHS and QoL scores was 4.8 months versus 2.8 months in patients treated with SoC (HR = 0.79, 95% CI, 0.59 to 1.05), resulting in a trend toward prolonged time to deterioration (TTD) with pembrolizumab versus SoC (85).

In the CheckMate 141 study, nivolumab also demonstrated a delay in clinically meaningful deterioration according to EORTC QLQ-C30, the absence of clinically meaningful worsening at week 15 according to EORTC QLQ-H&N35, and a clinically meaningful improvement from baseline to week 15 on the EQ-5D visual analog scale, in contrast to a clinically meaningful deterioration in the SoC group (86).

The use of ICI (pembrolizumab or nivolumab) as monotherapy in patients either in the first line or in further lines is an effective option that allows to avoid significant toxicities related to chemotherapy and discontinuation of treatment. Although characterized by toxicity, immunotherapy ensures high standards of quality of life.

### 5.1 Nutritional status

Most patients with head and neck cancer have weight loss, as their nutrition is often compromised due to many factors, such as disease, surgery, radiotherapy, and systemic cytotoxic treatment (87). Nutritional status is a key part of the oncology examination. In addition to measuring basic parameters such as body weight, weight change over the past few months, and PS ECOG, during each visit, it is necessary to focus on the signs and symptoms that may be the cause of the patient’s malnutrition such as dysphagia, mucositis, fatigue, and xerostomia.

There are many tools to assess the state of malnutrition, and none prevails over the others.

Among them, one of the most widely used is the Malnutrition Universal Screening Tool (MUST). This tool is quick and easy to use, and it has been shown to have clinical benefits in identifying patients with a risk of malnutrition early and receiving nutritional intervention (Malnutrition Advisory Group (MAG)) (88).

Nutritional problems begin with disease onset, with several studies suggesting that 25%–65% of head and neck cancer (HNC) patients present with malnutrition, while during treatment, it reaches 80% of cases (89) (90). Malnutrition is defined as more than 10% weight loss from normal body mass over 6 months or 5% weight loss over 3 months. Patients with a malnutrition status have a higher risk of infection, a poor quality of life, and a decrease in overall survival (91).

Nutritional status, before, during, and after the treatment, is highly recommended by international guidelines. When possible, oral food intake is preferred over enteral and parental nutrition. Resting energy expenditure (REE) measures the amount of total energy consumed at rest necessary to maintain vital physiological functions, and in patients with head and neck cancer, REE is approximately 22 kcal·kg^−1^·day^−1^ (92).

To preserve adequate nutritional support, current European Society for Clinical Nutrition and Metabolism (ESPEN) guidelines recommend an intake of 35 kcal·kg^−1^·day^−1^ and ≥1.5 g protein·kg^−1^·day^−1^ (93).

When the ability to eat is partially impaired, a semi-liquid diet combined with an oral nutritional supplement (ONS) is necessary. There are different formulations of ONS, but there are features that must be followed. They must have a high protein content and preferably also contain leucine and omega-3 fatty acids, helpful in preventing cachexia (94). ONS needs high energy density (2 kcal/mL) to increase patient compliance. Also, in this type of case, it is important for the patient to have small meals many times a day.

In cases where the patient is unable to eat, treatment is enteral or parenteral feeding. Enteral nutrition is preferred over parenteral because it avoids atrophy of the gastrointestinal tract, causes fewer infectious complications, and also reduces hospital length of stay. There are several methods for enteral feeding, but the most common is PEG.

The nasogastric tube (NGT) is used for a short period, usually less than 4 weeks, and is cheap and manageable. NGTs are used in patients with conserved airway reflexes who need enteral feeding for less than 30 days, and PEG is currently the “gold standard” for medium- to long-term enteral feeding for more than 30 days (95).

In patients with severe gastrointestinal tract dysfunction, the only option for nutritional support is the intravenous route. In oncology, parenteral nutrition (PN) is usually used in very advanced stage and end-of-life patients. It has to be introduced slowly, starting with 15–20 calories per kg of body weight per day with a maximum of 1,000 calories per day. PN carries the risk of potentially severe complications, including catheter-related infection, occlusion and thrombosis, electrolyte imbalance, and hepatopathy. Therefore, the indication for parenteral nutrition must be taken on a case-by-case basis under the judgment of the multidisciplinary team (96).

## 6 Biomarker

Immunotherapy is a key weapon in the treatment of metastatic/recurrent head and neck cancers, but only 20%–30% of patients have long-term benefits. The discovery of biomarkers that can predict immunotherapy response represents a major challenge in cancer research.

### 6.1 PD-L1

There are different scoring algorithms for PD-L1 staining: the TPS is a PD-L1 measurement in which only membranous staining of tumor cells is regarded as a significant staining. In contrast, the combined positive score (CPS) and inflammatory cell scoring (ICS) include and are restricted to PD-L1 expression in certain inflammatory cells, respectively.

Several trials have used TPS, among them CheckMate 141; in the prespecified exploratory analysis of a subgroup of patients with a PD-L1 expression level of 1% or more (57%), nivolumab provided OS benefit with a 45% reduction in the risk of death (HR = 0.55; 95% CI, 0.39 to 0.78). In PD-L1 non-expressors, nivolumab demonstrated a lower efficacy, with a 27% reduction in the risk of death compared with SoC (HR = 0.73; 95% CI, 0.49 to 1.09) (22). In exploratory qualitative immune profile analysis, the percent of PD-L1+ immune cells in the tumor microenvironment was associated with a higher median OS and greater likelihood of response to nivolumab *vs.* SoC (Cancer Research 2017) (97).

The KEYNOTE-040 used both CPS and TPS to assess PD-L1 expression, showing different HR in OS. In the intention-to-treat population, HR was 0.80 (0.65–0.98; p = 0.0161); among patients with PD-L1 CPS ≥ 1%, HR was 0.75 (0.59–0.95, p =0.0078); among patients with PD-L1 TPS ≥ 50%, HR was 0.54 (0.35–0.82, p = 0.0017) (23).

In phase III KEYNOTE-048, efficacy data correlate with PD-L1 expression and support the use of CPS as the optimal biomarker. Pembrolizumab monotherapy significantly improved OS in the PD-L1 CPS ≥ 20 (HR = 0.61) and CPS ≥ 1 (HR = 0.74) populations and led to non-inferior OS in the total population (HR = 0.81). Pembrolizumab–chemotherapy significantly improved OS in the PD-L1 CPS ≥ 20 (HR = 0.62), CPS ≥ 1 (HR = 0.64), and total populations (HR = 0.71) compared with cetuximab–chemotherapy (9). In *post-hoc* subgroup efficacy analyses of the PD-L1 CPS < 1, neither pembrolizumab monotherapy nor pembrolizumab–chemotherapy demonstrated improvement in OS over cetuximab–chemotherapy (HR = 1.51 and 1.21, respectively) (10).

In addition, attempts have been made to increase the reliability of PD-L1 expression detection through artificial intelligence technologies. Puladi et al. conducted a study using a novel approach with three sequentially applied neural networks for a fully automated assessment of PD-L1. Three PD-L1 scores were assessed: TPS, CPS, and ICS. This approach was validated using whole slide imaging (technology in which pieces of histologic tissues are scanned to produce digitized images) of HNSCC cases and compared with manual scoring of PD-L1 performed by human researchers. The inter-rater correlation (ICC) between humans and machine was very similar to the human–human correlation. The ICC was slightly higher in human–machine compared to human–human for the CPS and ICS but slightly lower for the TPS because human–human concordance was excellent for the TPS (98).

Nowadays, artificial intelligence applied to the measurement of PD-L1 in HNSCC tumors does not seem to be useful; further studies, are needed to account for operator-dependent heterogeneity in CPS assessment.

Another important topic is the temporal and spatial heterogeneity of CPS. In the 2021 European Society for Medical Oncology (ESMO) abstract, S.J. De Keukeleire presented data about biopsies in the primary tumor and metastatic site (lymph nodes or distant metastasis), and the discordance of CPS was approximately 34%. Recently, P. Bossi et al. analyzed the differences in CPS value in the primary tumor versus the metastatic site. Biopsies were taken in 56 patients either on the primary tumor or on the metastatic site (local or distant recurrence), and there was a concordance of CPS of 66%. These results are very similar, confirming a discordance about CPS PD-L1 expression of 33% between the primary tumor and the metastatic site (99).

Expression of PD-L2, the other ligand of PD-1, could be another potential biomarker of response to anti-PD-1 therapy. KEYNOTE-012 demonstrated that PD-L2 protein expression is correlated with a higher response to anti-PD-1 therapy (in terms of response rate), independently from PD-L1 expression (100).

### 6.2 HPV

Several preclinical studies showed how HPV-positive tumors correlate with a better prognosis and a better response to ICI, mainly due to an immunologically “warm” microenvironment.

In the CheckMate 141 study, regardless of the p16 status, the survival in the therapy arm with nivolumab was significantly longer (22). The single-arm phase II HAWK study evaluated durvalumab as monotherapy in platinum-refractory patients. In this study, an increase in ORR, PFS, and OS was demonstrated in HPV+ patients (101). In contrast, in KEYNOTE-040, HPV− cancers appeared to experience greater benefit from pembrolizumab (OS: HR = 0.77; CI, 0.61 to 0.97) rather than HPV+ cancers (23).

A pooled analysis of four studies (CheckMate 141, KEYNOTE-012, KEYNOTE-055, and HAWK) with a total of 425 patients showed that OS and ORR were better in HPV-positive patients than HPV-negative patients using PD-1/PD-L1 inhibitors (OS: HR = 0.71, p = 0.02; ORR: OR = 1.79, p = 0.01). Moreover, HPV-positive HNSCC patients exhibited greater T-cell infiltration than HPV-negative patients (p = 0.003) (102).

Due to the conflicting evidence regarding HPV’s role as a predictive biomarker for immunotherapy, HPV infection is not used in clinical practice as a predictive biomarker.

### 6.3 Tumor mutational burden

Tumor mutational burden (TMB), referred to as the sum of somatic mutations in cancer DNA with the following antigens recognized and targeted by the immune cells, is used as a biomarker for immunotherapy in different cancer types, especially in NSCLC.

A clear trend toward decreasing hazard ratio of death with increasing TMB cut-off was observed across cancer types demonstrating increasing benefit from ICI with higher TMB. Stratified analysis by selecting the higher mutation load quintile (top 20%) performed in different tumors stated that the TMB cut-point of HNSCC was 10 mut/Mb (103).

In a retrospective analysis of the EAGLE trial, the TMB was evaluated in plasma samples before treatment. This analysis showed that patients who have TMB values >16 mut/megabase benefit more in terms of OS from immunotherapy (with durvalumab or durvalumab and tremelimumab compared with chemotherapy). In contrast to patients who had low TMB (<16 mut/megabase), a clear benefit of immunotherapy versus chemotherapy was not evident. In the comparison of durvalumab *vs.* chemotherapy, OS HR was 0.39 in patients with blood TMB (bTMB) ≥ 16 and 0.91 in patients with bTMB < 16. The bTMB was independent of other clinical and prognostic factors such as HPV status, PD-L1 expression, age, gender, tumor location, and ECOG performance score (25).

This evidence indicates that high TMB predicts improved benefit from checkpoint inhibition in HNSCC, but so far, there is not yet consensus about a definitive threshold. At the moment, TMB testing in HNSCC is not recommended by the FDA and EMA.

## 7 The emerging role of target and combination therapy

With the increase of knowledge of molecular characterization of HNSCC, several studies have demonstrated the efficacy of target therapy individually or in combination with current standard treatments (**Table 2**).

**Table 2:** Summary characteristics of cited studies in target therapy.

Targeted agents		
Class	Drug or molecule	Key findings
EGFR	Cetuximab/nivolumab	Phase II study, median OS for Cohort A (prior therapy for R/M HNSCC), 11.4 months; median OS for Cohort B (not prior therapy), 20.2 months (104)
	Cetuximab/durvalumab	Phase II study, ORR 39% (105)
	Erlotinib/bevacizumab Cetuximab sarotalocan	Phase I/II study, ORR 15%; median PFS and OS of 4.1 and 7.1 months, respectively (106) Phase I/II ORR 28%, median PFS 5.7 months and OS 9.3 months (107)
HRAS	Tipifarnib	Phase II study, ORR 55%; median PFS 5.4 months; OS 15.4 months (108)
mTOR	Temsirolimus/cetuximab *vs.* temsirolimus	Phase II study, no difference for median PFS (TC arm, 3.5 months; T arm, 3.5 months) (109)
VEGFR	Axitinib	Phase II study, ORR 42% (75% for pts with mutations in the PI3K pathway and 17% for wild-type pts) (110)
	Lenvatinib/pembrolizumab	Phase I/Ib study (22 pts.); ORR 46%; median PFS 4.7 months (111)
PI3K	Buparlisib *vs.* placebo/paclitaxel	Phase II study (158 pts.); ORR 31%; median OS and PFS 10.4 and 4.5 months, respectively, in buparlisib arm (112)
HGF	Ficlatuzumab/cetuximab *vs.* ficlatuzumab	Phase II study; median PFS (combination arm 3.7 months); ORR 19% (113)
TGF-β and EGFR	BCA 101/pembrolizumab	Phase I/Ib study, ORR (in ITT population 48% (15/31), in HPV-negative patients 65% (13/20))
Nectin-4	Enfortumab vedotin	Phase II study, ORR 23.9%; median PFS 3.9 months; OS of 5.9 months (114)
Tissue factor	Tisotumab vedotin	Phase II study, ORR 40% (115)
Other immune checkpoint inhibitor combinations		
IDO	Epacadostat/pembrolizumab	Phase II study, ORR 34%, DCR 61% (116)
NK2GA	Monalizumab/cetuximab	Phase II study; ORR 36% in immunotherapy naive, 17% in immunotherapy pretreated (117)
HPV16 vaccine	PDS0101/pembrolizumab	Single-arm phase II study, median PFS 10.4 months, 12-month OS rate 87.1% (118)
T-cell exhaustion (LAG-3)	Eftilagimod alpha/pembrolizumab	Phase II study (36 pts.); ORR 36% (119)

HNSCC, head and neck squamous cell carcinoma; ORR, objective response rate; PFS, progression-free survival; OS, overall survival; DCR, disease control rate; HGF, hepatocyte growth factor; ITT, intention to treat; EGFR, epidermal growth factor receptor; R/M, recurrent/metastatic; HPV, human papillomavirus.

### 7.1 EGFR

Among the main targets, epidermal growth factor receptor (EGFR) is overexpressed in 80%–100% of head and neck squamous cell carcinomas. Significantly amplified EGFR occurred primarily in HPV-negative patients (120).

In a phase II trial, Chung et al. investigated the impact on OS of the nivolumab + cetuximab combination in patients with R/M HNSCC following the evidence about the release of interferon (IFN)-gamma and chemokines from natural killer (NK) cells after binding cetuximab to EGFR with the subsequent increase of PD-L1. The median OS in the 45 patients of Cohort A (who had prior therapy) was 11.4 months, with a 1-year OS of 50% (90% CI, 0.43 to 0.57), while the median OS in the 43 patients of Cohort B (who had no prior therapy) was 20.2 months, with a 1-year OS of 66% (90% CI, 0.59 to 0.71). This doublet could be a powerful strategy in this setting of disease in both I and II lines (104).

A combination of durvalumab and cetuximab was recently evaluated in a single-arm, phase II, non-randomized trial in patients with R/M HNSCC in the second line. ORR, the primary endpoint, was 39%, and the benefit was independent of PD-L1 expression (105).

In addition to monoclonal antibodies, several researchers focused on small molecules, such as tyrosine kinase inhibitors (TKIs) of EGFR, which are ineffective in HNSCC, although early results from other trials with combination therapies were promising. Erlotinib, for example, demonstrated modest improvements in PFS when used in combination with an anti-VEGF antibody (bevacizumab) in R/M HNSCC (106).

### 7.2 RAS

Braig et al. observed that RAS-activating mutations (HRAS/KRAS) are not very common in patients with cetuximab-naive HNSCC, while after treatment with cetuximab, one-third of patients developed acquired mutations in KRAS, NRAS, and HRAS. Furthermore, these were detected only in half of patients progressing to treatment, suggesting that the selective pressure exerted by cetuximab on tumor cells could determine the onset of the aforementioned resistance mutations responsible for disease progression (121).

Mutations in the HRAS (mHRAS) proto-oncogene occur in 4%–8% of patients with R/M HNSCC. L. Ho et al., in a single-arm, open-label, phase II trial, demonstrated the encouraging efficacy of tipifarnib, a farnesyltransferase inhibitor that disrupts HRAS function, in patients with R/M HNSCC with mHRAS variant allele frequency (VAF) of ≥20% (high VAF). In particular, ORR for patients with high VAF was 55%, and the median OS was 15.4 months (95% CI, 7.0 to 29.7) (108).

Data from 50 patients with high VAF were presented at the ESMO 2023 congress. The ORR in these patients was 30%, with one patient in complete response (CR). The most frequent grade 3 side effects (38%) were related to bone marrow toxicities, neutropenia (24%), anemia (20%), and leukopenia (14%) (122).

### 7.3 PI3K/AKT/mTOR and small TKI anti-VEGFR

Genomic alterations in one of the major components of the PI3K/AKT/mTOR pathway (e.g., PI3KCA, AKT1/2/3, and PTEN) were instead found in approximately 66% of HNSCC tumors and are also responsible for the development of resistance to the anti-EGFR therapy. PTEN loss might be part of a signature characteristic for resistance, as this may lead to compensatory activation of the PI3K/AKT pathway (123). To overcome these resistance mechanisms, the combination of PX-866, an oral PI3K inhibitor, with cetuximab was analyzed in 83 patients with advanced, platinum-refractory HNSCC who had received at least one, but no more than two, prior systemic treatments. Despite encouraging preclinical results, combined treatment was not superior to cetuximab monotherapy in terms of PFS (80 days *vs.* 80 days), OS (211 days *vs.* 256 days), and RR (10% *vs.* 7%) (124). The randomized phase II MAESTRO trial investigated the efficacy of temsirolimus, an mTOR inhibitor, with or without cetuximab. The study did not meet its primary endpoint (PFS), showing limited clinical activity of the combination in HNSCC R/M patients (109). Although co-targeting PI3K/mTOR and EGFR could be supported by inhibition of this pathway, preventing resistance to EGFR inhibitors, this combination has a severe toxicity profile and needs further investigation.

It is also known that tumors with PI3K alterations often induce angiogenesis through VEGF-regulated cytokine mechanisms. Swiecicki et al. demonstrated that treatment with axitinib, a potent inhibitor of VEGFR2, VEGFR3, and PDGFR, was associated with a relative response rate of 75% in patients with mutations of the PI3K pathway and 17% in wild-type patients (6 of 8 patients *vs.* 2 of 12 patients) (110). This is also the first study demonstrating that the targeted oral drug axitinib improves survival in patients with R/M HNSCC heavily pretreated: the overall survival rate at 6 months was 71%, while the median PFS and median OS were 3.5 months and 9.8 months, respectively.

Preclinical and clinical evidence suggests the possibility of enhancing the immunotherapeutic effectiveness of immune checkpoint inhibitors by modulating VEGF-mediated immune suppression through angiogenesis inhibition. In a phase I/Ib trial, the combination of pembrolizumab and lenvatinib was also investigated. The ORR was 46% (10/22 patients), and the median PFS was 4.7 months among the 22 patients with HNSCC (111).

Buparlisib is a pan-PI3K inhibitor and was evaluated alone and in combination with paclitaxel in a phase II randomized study (BERIL-1) in patients with platinum-pretreated recurrent metastatic HNSCC. It showed an ORR of 31% in the buparlisib group with a median PFS and OS of 4.5 and 10.4 months, respectively, compared with 3.5 and 6.5 months in the placebo group, regardless of PI3KCa mutations (112).

### 7.4 IDO-1

The IDO1 enzyme may be upregulated by tumors as a means of evading immune surveillance. A strong and extremely specific IDO1 enzyme inhibitor is epacadostat. Epacadostat plus pembrolizumab demonstrated an ORR of 34% and a disease control rate of 61% in ECHO-202/KEYNOTE-037; despite this result, the phase II study was prematurely stopped because of underwhelming findings in other tumor types (116).

### 7.5 NKG2A

An antibody called monalizumab is designed to block NKG2A receptors on CD8+ T cells and natural killer cells that infiltrate tumors and boost the immune system against cancer cells. In a phase II trial, the combination of monalizumab and cetuximab resulted in an ORR of 36% in patients who had never had immunotherapy and 17% in those who had. The 12-month OS estimate was 44% (117). The phase 3 INTERLINK-1 trial evaluated monalizumab plus cetuximab *vs.* cetuximab alone in patients with recurrent or metastatic HNSCC who have previously been treated with platinum-based chemotherapy and PD-L1 inhibitors but failed to meet the endpoints (125).

### 7.6 LAG-3

Eftilagimod alpha is a soluble agonist of the protein encoded by the LAG-3 gene that binds to a subset of the major histocompatibility complex class II molecules, facilitating the activation of antigen-presenting cells (APCs) and the recruitment and activation of CD4 and CD8 T cells. With an ORR of 36%, a phase II study evaluated the activity of eftilagimod alpha with pembrolizumab in the second line (36 patients) and presented encouraging results (119).

### 7.7 Hepatocyte growth factor

Hepatocyte growth factor/cMet pathway activation is a resistance mechanism of EGFR inhibition. Multicenter, randomized, non-comparative phase II study evaluated ficlatuzumab, an anti-hepatocyte growth factor, with or without cetuximab in R/M HNSCC in patients refractory to platinum and pembrolizumab. The study reached its primary endpoint with a median PFS of 3.7 months and an ORR of 19% (6/32). Interestingly, the patients who had an objective response had HPV-negative status (113).

### 7.8 TGF-β and EGFR

Transforming growth factor-beta (TGF-β) is a potent inhibitor of cell proliferation in the early stages of cancer, while in advanced stages, it has an opposite effect, increasing progression and tumor aggressiveness (126).

This controversial effect is known as the “TGF-β paradox”. This mechanism remains unknown, but one possible explanation could be in the cross-talk between TGF-β and EGFR signaling. These two pathways have a synergistic effect and amplify the process of epithelial–mesenchymal transition, thus supporting the process of metastasis (127).

A phase I/Ib study is evaluating first-line treatment in patients with metastatic HNSCC with CPS > 1, the BCA 101, a bifunctional antibody designed to inhibit the EGFR and TGF-β in combination with pembrolizumab. The ORR in all populations was 48% (15/31), but the most promising finding is in the HPV-negative population with an ORR of 65% (13/20). The most common adverse event was acneiform rash present in 75% of the population. These data need further evaluation in randomized clinical trials, especially in the HPV-negative population (128).

### 7.9 Antibody–drug conjugate

Nectin-4 is a protein involved in cell adhesion and is highly expressed in HNSCC, particularly expressed in non-smoking and p16-negative patients. Interestingly, nectin-4 expression was associated with a better prognosis (129). Enfortumab vedotin (EV) is an antibody–drug conjugate (ADC) directed against nectin-4 and is currently approved in the treatment of metastatic urothelial carcinoma. EV was evaluated in a phase II basket study assessed in various types of pretreated metastatic solid tumors. Among them, the HNSCC cohort was 44 patients, and most had received more than two lines of therapy in the metastatic setting. The ORR was 23.9%, and the disease control rate (DCR) was 56.5%. The most common side effects were skin reaction (43%), peripheral neuropathy (32.6%), and hyperglycemia (4.3%). These results are encouraging and need further investigation (114).

HER3 is responsible for aberrant activation of PI3K/mTOR signaling and is one of the mechanisms of resistance to therapy against EGFR; moreover, its overexpression is associated with a worse prognosis across solid tumors (130). In a phase I study, Zhang et al., in various heavily pretreated metastatic solid tumors, evaluated BL-B01D1, a conjugated bispecific antibody directed against EGFR/HER3 and linked to a topoisomerase I inhibitor. The cohort of patients with HNSCC was 13 patients, with an ORR of 7.7%. The most frequent side effects were bone marrow toxicity (including leukopenia in 60%), alopecia in 30%, and vomiting in 28% (131).

Tisotumab vedotin (TV) is a conjugated antibody directed against tissue factor, currently approved for the treatment of metastatic cervical cancer. In the interim analysis of InnovaTV 207 study, a multicenter phase IIb study evaluating TV for advanced tumors, including patients with R/M HNSCC. The HNSCC cohort consisted of 15 heavily pretreated patients with at least containing platinum and checkpoint inhibitors. The ORR was 40% (95% CI, 16.3 to 67.7), with one complete response and five partial responses. Side effects were manageable, and the most frequent were asthenia and peripheral neuropathy. To date, the trial is still enrolling (115).

Cetuximab sarotalocan is an ADC directed against EGFR and bound to a light-activatable dye. Preclinical research shows that activation of the dye with non-thermal red light (690 nm) results in rapid antitumor action driven by biophysical processes that alter cell membrane integrity (132). The phase IIa study evaluated the antitumor activity of sarotalocan cetuximab in 30 heavily pretreated R/M HNSCC patients. Twenty-four hours after infusion administration of the drug, non-thermal red light was used to illuminate tumor areas. The ORR was 28%, and the median PFS and OS were 5.7 months and 9.3 months, respectively. The most common side effect of grade ≥ 3 was skin reaction (18%) and paronychia cracking (12%) (107). From these results, cetuximab sarotalocan has been approved by the Pharmaceuticals and Medical Devices Agency (PMDA) of Japan for the treatment of locally advanced or recurrent unresectable HNSCC. In Western countries, it is not yet approved, and further investigations are needed.

### 7.10 HPV16 vaccine

PDS0101 is a vaccine composed of neoantigens of liposomal E6/E7 HPV16, leading to a polyclonal expansion of HPV16-specific CD8 and CD4 T cells, and exhibits antitumor activity in combination with checkpoint inhibitors through upregulation of type I interferons and promotion of antigen processing and presentation. In the phase 2 VERSATILE-002 study, PDS0101 and pembrolizumab were used to treat patients with recurrent or metastatic HPV16-related HNSCC. The results were discussed at the 2023 American Society of Clinical Oncology (ASCO) annual meeting. Among the 48 patients naive to checkpoint inhibitor therapy (ICI naive), nine had a partial response (including complete response), and 15 had stable disease. The median PFS was 10.4 months, and the estimated overall survival at 12 months was 87.1%. These promising results are under investigation in a confirmatory phase III trial (NCT04260126) (118).

## 8 Future perspectives

The TME plays a key role in promoting all “hallmarks of cancer” (133). Cancer-associated fibroblasts (CAFs) are a component of the TME, are responsible for the production of the extracellular matrix (ECM), and contribute to an extremely complex network of connections between various cells in the microenvironment and cancer cells. Interestingly, CAFs can modulate the immune system through several mechanisms (134).

The release of cytokines by CAFs is responsible for the “corruption” of macrophages into tumor-associated macrophages (TAMs) that help generate an immunosuppressive state. CAFs also strongly interfere with NK cells, particularly through the production of cytokines that inhibit NK cell cytotoxicity. Through the release of TGF, CAFs induce T-cell apoptosis (135).

According to Sasaki (2018) (136), CAFs may also play a role in the formation of a fibrous capsule surrounding tumors, and this may block the process of migration and infiltration of T lymphocytes toward the tumor.

One of the most important pathways regulating the differentiation of fibroblasts into CAFs is the NOX pathway, a group of enzymes that play an important role in the cellular stress response through the production of reactive oxygen species (ROS) (137).

The antitumoral activity of setanaxib, a potent inhibitor of NOX4 and NOX1 isoforms, is under investigation in a multicenter, randomized, phase II trial for R/M HNSCC patients with a CPS score >1 and a positive level of CAF (defined as a level of CAF in ≥5% in the tumor) in combination with pembrolizumab (NCT05323656).

## 9 Conclusions

The treatment of head and neck cancer is a tough challenge for clinicians. The holistic approach to a frail patient like the one affected by R/M HNSCC is based on the support of new different professional figures such as nutritionists, dentists, molecular pathologists, pain therapy specialists, and psychologists who can cooperate with traditional surgeons, radiotherapists, and medical oncologists. Teamwork is the prelude to proper treatment planning according to biological and clinical evaluation. Only by following this strategy will it be possible to identify the correct frame within which to attribute the best setting for each patient. In this context, the integration of systemic and locoregional treatments is critical in order to answer the needs of a single patient with either symptomatic or curative intent. Radiotherapy and salvage surgery are the only curative treatment choices in patients with locoregional recurrence and should be considered in high-volume and highly specialized centers. Palliative radiotherapy has a significant role in improving the patient’s symptoms, and several ongoing studies allow a de-escalation of the radiation with a reduction of toxicities.

After decades of standard chemotherapy characterized by limited activity and a high toxicity profile, the appearance of cetuximab first and the immunotherapy later significantly improved the outcome of these patients. Despite these encouraging results, there are still important questions regarding the identification of predictive and prognostic factors (what does the future hold after PD-L1)? and the correct combination or sequences of available tools. Until now, no prospective data about the activity of systemic treatments after immunotherapy have been published. Although data about molecular profiling are available, there is poor evidence regarding the activity of new target therapies. At the moment, the cornerstone of treatment in R/M HNSCC patients derives from the KEYNOTE-048 phase III trial, which demonstrated the significant role of immunotherapy either in combination or alone in patients sensitive to ICI. This study offered the possibility of an active treatment to patients not suitable to be treated with chemotherapy. Chemotherapy in combination with cetuximab or with immunotherapy is still the best option for patients who need tumor shrinkage because of early metastatic or symptomatic disease. Quality of life is one of the main topics in this category of frail patients, and we hope that the new scientific knowledge will allow us to improve not only OS but also this important clinical aspect.

## Author contributions

SP: Conceptualization, Data curation, Formal analysis, Investigation, Methodology, Resources, Validation, Visualization, Writing – original draft, Writing – review & editing, Software. AD: Conceptualization, Data curation, Formal analysis, Investigation, Methodology, Resources, Visualization, Writing – original draft, Writing – review & editing, Software. DO: Data curation, Formal analysis, Investigation, Methodology, Resources, Software, Visualization, Writing – original draft, Writing – review & editing. AS: Data curation, Formal analysis, Investigation, Methodology, Resources, Visualization, Writing – original draft, Writing – review & editing. GM: Data curation, Formal analysis, Investigation, Methodology, Resources, Visualization, Writing – original draft, Writing – review & editing. GV: Data curation, Formal analysis, Investigation, Methodology, Resources, Visualization, Writing – original draft, Writing – review & editing. MQ: Formal analysis, Supervision, Writing – review & editing. MD: Formal analysis, Supervision, Writing – review & editing. GT: Formal analysis, Funding acquisition, Supervision, Validation, Writing – review & editing. AC: Conceptualization, Data curation, Formal analysis, Funding acquisition, Investigation, Methodology, Project administration, Resources, Supervision, Validation, Visualization, Writing – original draft, Writing – review & editing.
